# A case of special basket impaction removal during endoscopic retrograde cholangiopancreatography using biopsy forceps under choledochoscope

**DOI:** 10.1055/a-2489-8058

**Published:** 2024-12-17

**Authors:** Jinxin Li, MengQiang Cai, YuRong Cui, Bing Zhao, Junying Liu

**Affiliations:** 1 537323 Department of Gastroenterology, The First Affiliated Hospital of Henan University of Traditional Chinese Medicine, Zhengzhou, China; 2 87803 Department of Gastroenterology, The First Affiliated Hospital of Hunan Normal University, Zhengzhou, China

A 72-year-old man who presented with fever, chills, abdominal pain, and jaundice was admitted to the hospital. He had a history of bile duct cancer and underwent laparoscopic gallbladder-jejunostomy for biliary obstruction two years ago. Two ventricular drainage catheters with a diameter of 12 Fr were placed through the gallbladder into the right and left intrahepatic bile ducts. Preoperative magnetic resonance cholangiopancreatography (MRCP) revealed there were filling defects in the common hepatic duct and the upper end of the common bile duct, suspected to be stones, and the catheterization shadows from the gallbladder to the common bile duct were observable ( Fig. 1 ). The decision to perform endoscopic retrograde cholangiopancreatography (ERCP) for stone removal was made after a multidisciplinary discussion.

**Fig. 1:**
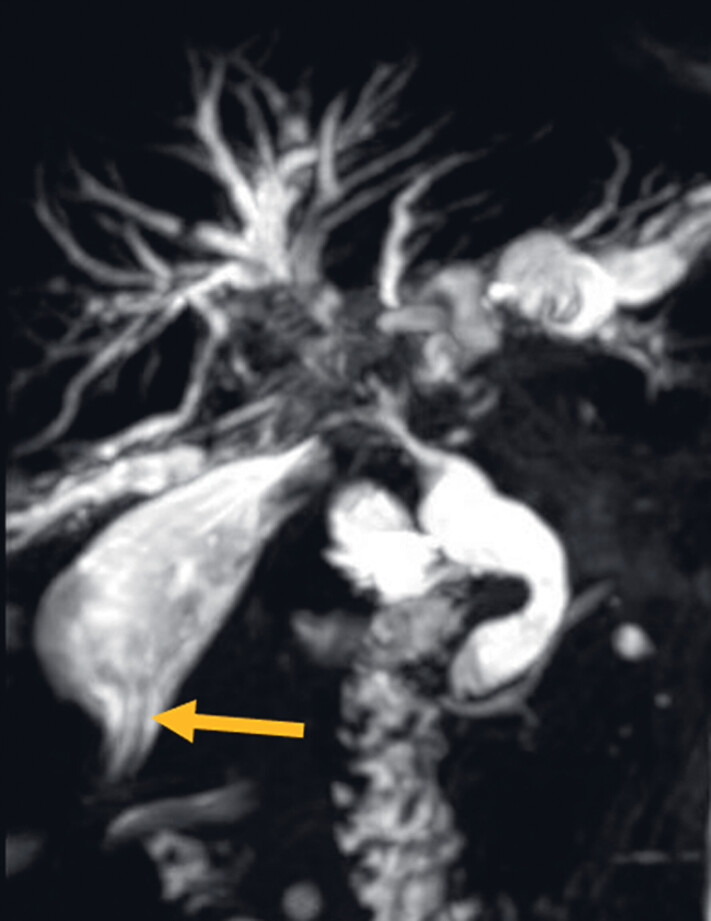
Preoperative magnetic resonance cholangiopancreatography (MRCP) revealed the shadow of the internal duct associated with the gallbladder (indicated by the yellow arrow).

During the stone extraction process, it was observed that the basket (FG-V432P; Olympus, Tokyo, Japan) was impacted by the drainage catheter ( Fig. 2 ). Following exploration with a choledochoscope (M00546600; Boston Scientific, Marlborough, Massachusetts, USA), we used biopsy forceps (M00546470; Boston Scientific) to gradually sever the drainage catheter, successfully resolving the impaction ( Video 1 ). Subsequently, a nasal biliary catheter (L14725D; Leo Medical Co., Ltd., Changzhou, China) was inserted into the left intrahepatic bile duct to drain the bile and contrast medium. Four days post-operation, during gastroscopy, the nasal biliary catheter was transected at the duodenal bulb using an endoscopic scissor (JHY-FG-23-230-A6; Jiuhong Medical, Changzhou, China), converting its remainder into a stent. Bile drainage proceeded smoothly thereafter ( Fig. 3 ).

**Fig. 2:**
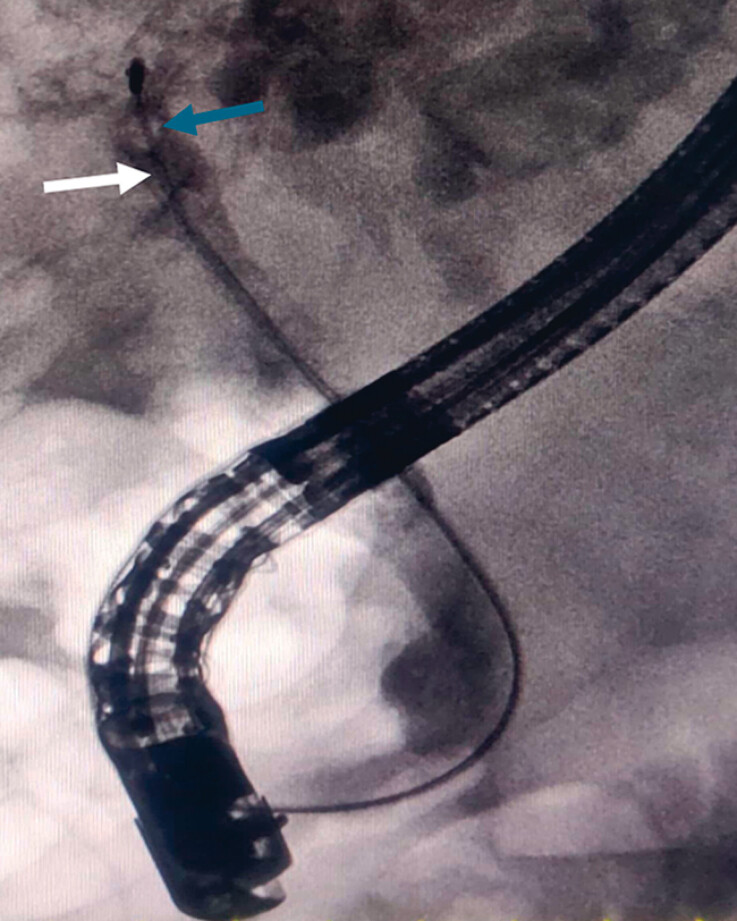
X-ray imaging revealed the impaction of the basket (indicated by the white arrow), along with the cross-section of a drainage catheter (indicated by the blue arrow).

**Fig. 3:**
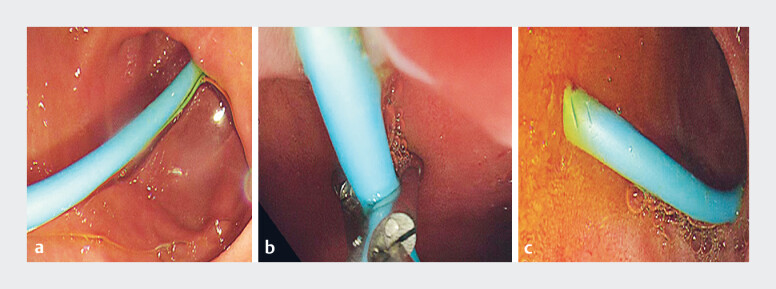
**a** Gastroscopy revealed that the nasal bile duct was not displaced. **b** The nasal bile duct was subsequently severed. **c** The bile drainage remained unobstructed.

A case of special basket impaction removal during endoscopic retrograde cholangiopancreatography using biopsy forceps under choledochoscope.Video 1

Currently, in contrast to stone-related impaction, those associated with drainage catheters cannot be resolved using choledochoscope laser lithotripsy or extracorporeal shock wave lithotripsy (ESWL) 1 2 . The application of argon plasma coagulation (APC) for cutting the basket may lead to electrical burns, whereas surgical intervention tends to be more traumatic 3 . In this case, we cut off the drainage catheter under choledochoscope by using biopsy forceps, successfully released the impaction, and retrieved the basket without any adverse events. This may be an effective measure for drainage catheter-related impaction.

Endoscopy_UCTN_Code_CPL_1AK_2AF

## Notes

Endoscopy E-Videos https://eref.thieme.de/e-videos
*E-Videos* is an open access online section of the journal *Endoscopy* , reporting on interesting cases and new techniques in gastroenterological endoscopy. All papers include a high-quality video and are published with a Creative Commons CC-BY license. Endoscopy E-Videos qualify for HINARI discounts and waivers and eligibility is automatically checked during the submission process. We grant 100% waivers to articles whose corresponding authors are based in Group A countries and 50% waivers to those who are based in Group B countries as classified by Research4Life (see: https://www.research4life.org/access/eligibility/ ). This section has its own submission website at https://mc.manuscriptcentral.com/e-videos .
